# Private sector availability and affordability of under 5 malaria health commodities in selected states in Nigeria and the Federal Capital Territory

**DOI:** 10.1080/20523211.2023.2294024

**Published:** 2023-12-27

**Authors:** Kunle Rotimi, Babatunde Fagbemi, Ademola Joshua Itiola, Taiwo Ibinaiye, Adaeze Aidenagbon, Chrysantus Dabes, Ahmed Aminu Biambo, Azuka Iwegbu, Sarah Onabajo, Chibuzo Oguoma, Olusola Oresanya

**Affiliations:** a Malaria Consortium, Abuja, Nigeria; b West African Postgraduate College of Pharmacists, Lagos, Nigeria; c National Agency for Food and Drug Administration and Control, Lagos, Nigeria

**Keywords:** Access to medicines, availability, affordability, antimalarial commodities, pharmaceutical policy

## Abstract

**Background:**

To guarantee uninterrupted service delivery, quality-assured products must be affordable and continuously available across all sectors, including the private sector, which provides more than 60% of healthcare services in Nigeria. We investigated the private sector availability and affordability of under 5 malaria commodities to establish the level of access in this sector.

**Methods:**

We surveyed patent medicine and pharmacy stores across seven states in Nigeria and the Federal Capital Territory to establish the availability and affordability of selected malaria commodities for children under 5 years. Availability was measured as the percentage of visited outlets with the product of interest on the day of visit, while affordability was assessed by establishing if it cost more than a day’s wage for the least-paid government worker to purchase a full course of malaria diagnostic test and/or medication.

**Results:**

Artemisinin-based antimalarials for uncomplicated and severe malaria were the most available commodities. SPAQ1 and SPAQ2 used for seasonal malaria chemoprevention campaign were surprisingly also available in some outlets. However, only about half (48.3% and 53.3%) of the surveyed outlets had stock of artemether/lumefantrine (AL1) and artesunate injection, respectively. The median price of surveyed products ranged from USD (United States Dollars) 0.38 to USD 2.17 per treatment/test. Except for amodiaquine tablet and artemether injection, which cost less, all other originator brands cost the same or more than the lowest-priced generic. Antimalarial products were affordable as their median prices were not more than a day’s wage for the least-paid government worker. However, when the cost of testing and treatment with artemisinin-based combination therapies (ACTs) was assessed, testing and treatment with dihydroartemisinin/piperaquine were unaffordable as the they cost more than 1.5 times the daily wage of the least-paid government worker.

**Conclusion:**

The overall private sector availability of under-five malaria commodities in surveyed locations was suboptimal. Also, testing and treatment with recommended ACTs were not affordable for all surveyed products. These findings suggest the need for interventions to improve access to affordable under-five malaria commodities.

## Background

With nearly 95,000 annual deaths and more than 50 million annual cases among children under 5 years, malaria is a major public health challenge in Nigeria (Adejoro, 2023; Dasgupta et al., 2022; Okorosobo et al., 2011; World Health Organization, 2022a; Yusuf et al., 2010). Poor access to diagnostic and treatment services, including the associated health commodities, is a leading reason for the persistence of high malaria morbidity and mortality, suggesting that improving access both in the public and private sectors is critical to improving child survival (Dasgupta et al., 2022; Oladepo et al., 2019; Ugonna, 2014).

The private sector, which delivers more than 60 percent of healthcare services in Nigeria, is often overlooked when intervening to improve access, as most interventions focus on the public sector (Management Sciences for Health, 2021; Omogbolagun, 2021). Nearly 30 percent of households in Nigeria visit either a private pharmacy that are licensed to stock all categories of medicines under the supervision of a registered pharmacist or patent medicine store (PMS) private stores that are licensed to only stock and sell over the counter medicines for malaria services and other health conditions (Edwards et al., 2022a; Okonkwo & Okonkwo, 2010; Oyeyemi et al., 2020), highlighting the importance of these outlets in ensuring access to malaria diagnosis and treatment.

Studies suggest that the availability of malaria rapid diagnostic test (mRDT) in the private sector in Nigeria is suboptimal (22.1% in pharmacies versus 13.6% in patent medicine stores) with only slight improvement from 2014 to 2018 (Edwards et al., 2022a; Poyer et al., 2015). Antimalaria medicines, on the other hand, were on average more available, especially from 2010 to 2017 when two major projects, Affordable Medicines Facility-Malaria (AMFm) and private sector co-payment mechanism (PSCM) (Arnold et al., 2012; Edwards et al., 2022b; Tougher et al., 2012; Unitaid, 2021) aimed at working with manufacturers to improve production and supply of quality-assured malaria commodities in the private sector were implemented (Adeyi & Atun, 2010; Ye et al., 2015).

These prior studies on access to malaria commodities did not address the affordability of mRDT in the private sector (Edwards et al., 2022a; Poyer et al., 2015). They were mainly conducted before the COVID-19 pandemic, which significantly disrupted global supply chains (Kazancoglu et al., 2023; Moosavi et al., 2022). Also, no study has been conducted to ascertain if the gains recorded during the AMFm and PSCM era were sustained. Finally, continuous monitoring of access to malaria commodities is required to track progress towards universal health coverage (Joda et al., 2015); hence, more recent evidence is needed.

We therefore conducted this study to ascertain access to selected under 5 malaria commodities, focusing on two domains of access, availability and affordability in the private sector (Tougher et al., 2012; Unitaid, 2021). We also examined variation in availability and affordability by location (rural versus urban) and type of outlet (pharmacy versus patent medicine store).

## Methods

### Study area and sampled facilities

This study was conducted in seven Nigerian states and the Federal Capital Territory (FCT) (Table 1), where Malaria Consortium supported seasonal malaria chemoprevention (SMC) campaigns. Two patent medicine stores and one private pharmacy store were surveyed across all the local government areas (LGAs) in each state except in Oyo State, where only the six LGAs supported by Malaria Consortium were visited (Table 1). In total, we surveyed 127 pharmacies and 254 patent medicine stores.

**Table 1.** Number of facilities surveyed by state.

Study states	Number of commercial medicine outlets		
	Pharmacy	Patent medicine store	Total
Bauchi	20	40	60
FCT	6	12	18
Kogi	21	42	63
Kebbi	21	42	63
Nasarawa	13	26	39
Oyo	6	12	18
Sokoto	23	46	69
Plateau	17	34	51
**Grand Total**	**127**	**254**	**381**

### Data collection tool

The survey form was adapted from the WHO/HAI data collection tool for measuring medicine prices, availability and affordability (World Health Organization, 2012) and deployed on an electronic platform (surveyCTO).

### Data collection

Following a standardised protocol, trained data collectors (one per LGA) visited the selected private outlets. All the data collectors have at least a bachelor’s degree and have prior research/programme evaluation data collection experience. For each private outlet visited, data were collected on the availability and prices of both the lowest-priced generic (LPG) and the originator brand (OB) for selected malaria commodities (Table 2). Data were only collected for the LPG for products whose OBs were no longer available or could not be ascertained.

**Table 2.** List of malaria commodities surveyed.

Group	Indication	Generic name	Dosage form	Strength	Originator brand (OB)	Manufacturer
Artemisinin-based combination therapy (ACT) (first line treatment)	Uncomplicated malaria	Artemether/Lumefantrine (AL1)	Tablet	20 mg/120mg	Coartem	Novartis
		Artesunate/Amodiaquine (AA1)	Tablets	25 mg/67.5mg	Coarsucam	Sanofi
		Dihydroartemisinin/Piperaquine (DHP)	Tablet	20 mg/160mg	Guillin DHP	Guillin DHP
Artemisinin-based monotherapy		Artesunate tablet	Tablet	25mg	Artesunat	Mekophar
		Dihydroartemisinin (DHA)	Tablet	20mg	NA	NA
Non-artemisinin-based monotherapy		Chloroquine (CQ)	Syrup	50 mg/5ml	Nivaquine	May & Baker
		Quinine	Tablet	300mg	*NA	NA
		Quinine	Syrup	100 mg/5ml	*NA	NA
		Amodiaquine	Tablet	200mg	Camoquine	Pfizer
Artemisinin-based injectables	Severe malaria	Artesunate	Injection	30mg	Artesun	Guillin
		Artemether	Injection	20mg	Paluther	Sanofi
Non-artemisinin-based injectables		Chloroquine (CQ)	Injection	40 mg/ml	*NA	NA
		Quinine	Injection	300 mg/ml	*NA	NA
Non-artemisinin-based combination therapy	Chemoprevention	Sulphadoxine/Pyrimethamine (SP)	Tablet	500 mg/25mg	Swidar/Fansidar	SWIPHA
		Sulpadoxine/Pyrimethamine/Amodiaquine (SPAQ1)	Tablet	250 mg/12.5 mg/75mg	SPAQ-CO	Guillin
		Sulpadoxine/Pyrimethamine/Amodiaquine (SPAQ2)	Tablet	500 mg/25 mg/150mg	SPAQ-CO	Guillin
Diagnostic Tests	Diagnosis	Malaria rapid diagnostic test (mRDT)	Tests	NA	*NA	NA

NA: Not applicable; *Products whose originator brands are no longer available or cannot be ascertained.

### Outcomes

- Availability

The percentage of visited outlets with the product of interest on the day of visit.

ii. Affordability

The number of income days required to purchase (a full course of) malaria health products. This was estimated by dividing the median cost of the product(s) by the daily wage of the least-paid government worker. The product(s) is/are affordable if it/they do(es) not cost(s) more than a day’s wage for Nigeria’s least-paid government worker.

### Data analysis

Descriptive statistics in the form of counts and percentages were used to describe the availability of the OB (and LPG) for each malaria commodity. To establish affordability, we presented affordability as the ratio of the median price of malaria commodity(ies), the cost of individual malaria product or the combined cost of artemisinin-based combination therapy (ACT) and mRDT to a day’s wage of the least-paid government employee (daily wage = 1,000 Nigeria Naira (2.16 United States Dollar (USD)) as of December 2022). All costs were expressed in USD using the Central Bank of Nigeria’s (CBN) exchange rate of 1 USD to 461.50 Nigerian Naira as of May 27, 2023. In addition to the overall estimates of availability and affordability, we further stratified results by type of outlet (pharmacy versus patent medicine store), and location (rural versus urban).

All analyses were conducted using Microsoft Excel 2016 and R version 2016.

## Results

### Number of private outlets

**Figure 1.**
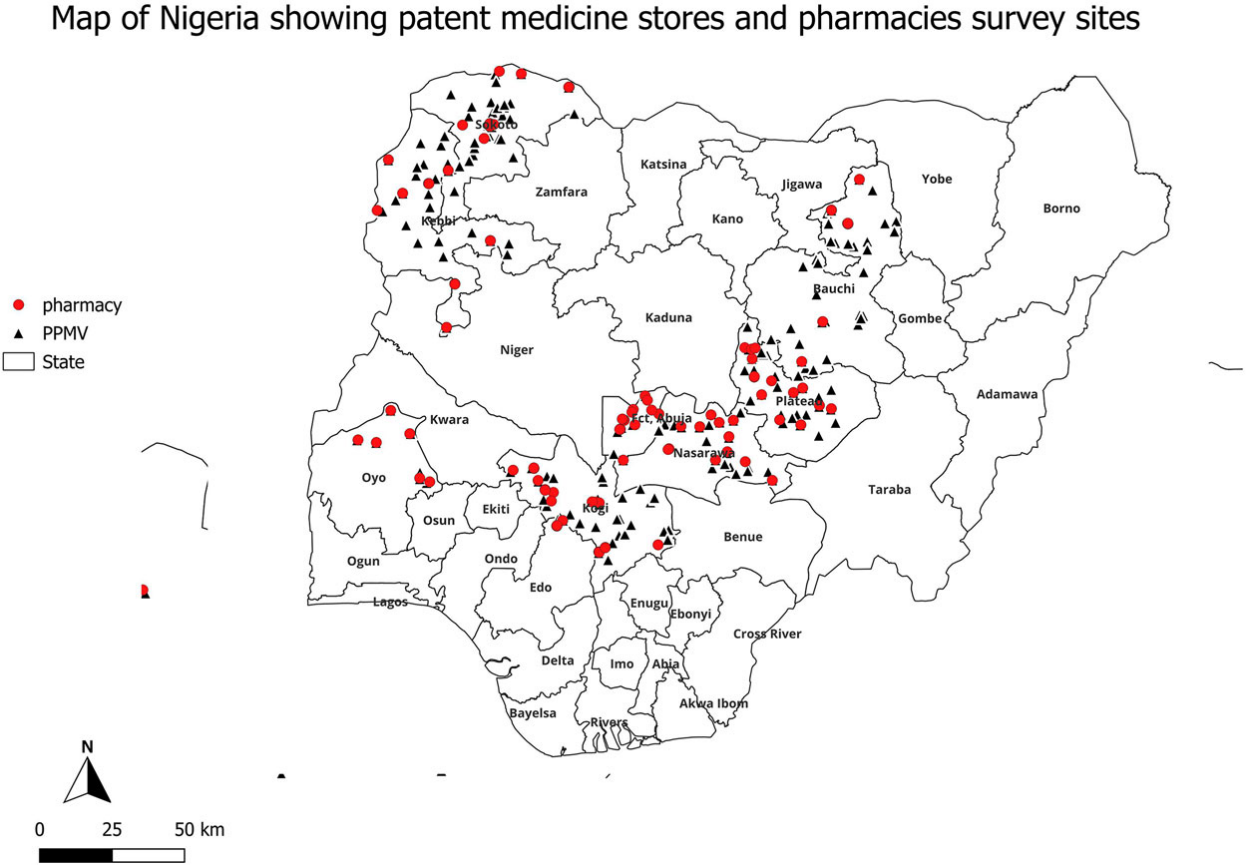
Map of Nigeria showing the surveyed outlets.

We surveyed a total of 381 private outlets comprising of 127 pharmacies and 254 patent medicines stores between December 2022 and January 2023. Figure 1 below shows the spread of surveyed sites across all states and the FCT.

### Private sector availability of under 5 malaria commodities

#### Overall availability

Across all products, the originator brands were more available than the lowest-priced generic. The originator brand of AL1 and artesunate injection were the most available antimalarials, with availability of 48.3% and 53.3%, respectively. The most available chemopreventive agent was the originator brand of sulphadoxine/pyrimethamine, with an availability of 27.3% while mRDT was available in 37.2% of visited outlets (Table 3).

**Table 3.** Overall availability of the selected malaria commodities.

Group	Commodities	OB (%)	LPG (%)
Artemisinin-based combination therapy (ACT) for uncomplicated malaria	AL1	48.3	36.5
	AA1	12.8	7.1
	DHP	19.0	5.9
Artemisinin-based monotherapy for uncomplicated malaria	Artesunate tab	13.3	9.2
	Dihydroartemisinin tab	N/A	9.5
Non-artemisinin-based monotherapy for uncomplicated malaria	Chloroquine syr	30.6	28.0
	Quinine tab	N/A	14.7
	Quinine syr	N/A	16.8
	Amodiaquine tab	8.1	6.6
Artemisinin-based injectables for severe malaria	Artesunate inj	53.3	19.2
	Artemether inj	45.7	39.3
Non-artemisinin-based injectables for severe malaria	Chloroquine inj	N/A	34.1
	Quinine inj	N/A	25.4
Non-artemisinin-based combination therapy for chemoprevention	SP	27.3	11.4
	SPAQ1	N/A	3.8
	SPAQ2	N/A	4.5
Diagnostics Tests	mRDT	N/A	37.2

inj: injection; N/A: Not applicable; tab: tablet.

#### Availability by location

When availability was stratified by location, the originator brand was still more available than the lowest-priced generic in both rural and urban areas, except for the urban availability of chloroquine syrup and amodiaquine tablets. For most products, availability was higher in the urban areas compared to rural areas except for the OBs of AL1, chloroquine syrup, amodiaquine tablet and artemether injection, as well as LPGs of amodiaquine tablet, SPAQ1, SPAQ2 and mRDT (Table 4).

**Table 4.** Availability of malaria commodities in rural areas versus urban areas.

Group	Commodities	Rural		Urban	
		OB (%)	LPG (%)	OB (%)	LPG (%)
First Line Treatment, ACT for uncomplicated malaria	AL1	48.8	35.6	47.7	37.8
	AA1	11.2	5.6	15.1	9.3
	DHP	12.8	4.8	27.9	7.6
Artemisinin- based monotherapy for uncomplicated malaria	Artesunate tab	13.2	8.8	13.4	9.9
	Dihydroartemisinin tab	NA	8.0	NA	11.6
Non-artemisinin-based monotherapy for uncomplicated malaria	Chloroquine syrup	33.2	27.6	26.7	28.5
	Quinine tab	NA	12.4	NA	18.0
	Quinine syrup	NA	12.0	NA	23.8
	Amodiaquine tab	9.6	6.8	5.8	6.4
Artemisinin based for severe malaria	Artesunate inj	42.8	14.8	68.6	25.6
	Artemether inj	46.0	36.0	45.3	44.2
Non-artemisinin based for severe malaria	Chloroquine inj	NA	28.8	NA	41.9
	Quinine inj	NA	22.0	NA	30.2
Non-artemisinin-based combination therapy for chemoprevention	SP	25.6	10.0	29.7	13.4
	SPAQ1	NA	4.4	NA	2.9
	SPAQ2	NA	6.0	NA	2.3
Diagnostic Test	mRDT	NA	37.6	NA	36.6

#### Availability by type of outlet

Similar to availability by location, the originator brand was more available than the lowest-priced generic in both pharmacies and patent medicines stores, except for the pharmacy availability of chloroquine syrup. The overall availability of OBs and LPGs was higher in pharmacies than in patent medicine stores. The only exception to this was the availability of LPGs of SPAQ1 and SPAQ2 (Table 5).

**Table 5.** Availability by type of outlet.

Group	Commodities	PMS		Pharmacy	
		OB (%)	LPG (%)	OB (%)	LPG (%)
First Line Treatment, ACT for uncomplicated malaria	AL1	48.1	34.3	49.0	43.3
	AA1	10.7	6.0	19.2	10.6
	DHP	13.2	4.4	36.5	10.6
Artemisinin- based monotherapy for uncomplicated malaria	Artesunate tab	11.3	8.2	19.2	12.5
	Dihydroartemisinin tab	NA	5.7	NA	21.2
Non-artemisinin-based monotherapy for uncomplicated malaria	Chloroquinine syrup	30.2	26.1	31.7	33.7
	Quinine tab	NA	10.1	NA	28.9
	Quinine syrup	NA	12.6	NA	29.8
	Amodiaquinine tab	6.6	5.7	12.5	9.6
Artemisinin based for severe malaria	Artesunate inj	49.4	15.7	65.4	29.8
	Artemether inj	44.0	37.1	51.0	46.2
Non-artemisinin based for severe malaria	Chloroquinine Inj	NA	30.2	NA	46.2
	Quinine inj	NA	18.9	NA	45.2
Non-artemisinin-based combination therapy for chemoprevention	SP	24.2	9.1	36.5	18.3
	SPAQ1	NA	4.1	NA	2.9
	SPAQ2	NA	5.4	NA	1.9
Diagnostic Test	mRDT	NA	35.9	NA	41.4

### Cost and affordability

#### Overall median price, median price ratio and affordability

The median price of surveyed products ranged from USD (United States Dollar) 0.38 to USD 2.17 per treatment/test. Except for amodiaquine tablet and artemether injection, which cost less, originator brands cost the same or more than the LPG (Table 6). All products were affordable as their median prices were not more than a day’s wage for the least-paid government worker (number of days of wage ≤1). Products require as low as 0.18 days of wage (Quinine injection) and as high as 1 day of wage DHP(OB) and Dihydroartemisinin tablet (LPG) for the least-paid government worker (Table 6).

**Table 6.** Median price, median price ratio and overall affordability of under 5 malaria health products.

Group	Commodities	Median Price (USD)		Median price ratio	Affordability: Number of days of wages	
		OB	LPG	OB: LPG	OB	LPG
First Line Treatment, ACT for uncomplicated malaria	AL1	0.87	0.87	1.00	0.40	0.40
	AA1	1.41	1.30	1.08	0.65	0.60
	DHP	2.17	2.06	1.05	1.00	0.95
Artemisinin- based monotherapy for uncomplicated malaria	Artesunate tab	1.52	1.08	1.40	0.70	0.50
	Dihydroartemisinin tab	NA	2.17	NA	NA	1.00
Non-artemisinin-based monotherapy for uncomplicated malaria	Chloroquine syrup	0.63	0.54	1.16	0.29	0.25
	Quinine tab	NA	1.08	NA	NA	0.50
	Quinine syrup	NA	1.52	NA	NA	0.70
	Amodiaquine tab	0.65	0.76	0.86	0.30	0.35
Artemisinin based for severe malaria	Artesunate inj	1.08	1.08	1.00	0.50	0.50
	Artemether inj	0.65	0.76	0.86	0.30	0.35
Non-artemisinin based for severe malaria	Chloroquine inj	NA	0.65	NA	NA	0.30
	Quinine inj	NA	0.38	NA	NA	0.18
Non-artemisinin-based combination therapy for chemoprevention	SP	0.65	0.65	1.00	0.30	0.30
	SPAQ1	NA	0.43	NA	NA	0.20
	SPAQ2	NA	0.43	NA	NA	0.20
Diagnostic Test	mRDT	NA	0.43	NA	NA	0.20

#### Median prices and affordability by location

Overall, most of the products either require the same days of wage (OB and LPG of AL1 and artesunate injection) or fewer in the rural areas compared to those in the urban areas. However, some products, artesunate tablet (OB and LPG) and originator brand of chloroquine syrup required fewer days of wage in the urban area compared to the rural area (Table 7). All products except for the originator brand of DHP (urban) were affordable.

**Table 7.** Median prices and affordability in urban and rural areas.

Groups	Products	OB				LPG			
		Rural		Urban		Rural		Urban	
		Median Prices	Days of wages	Median Price	Days of Wages	Median Prices	Days of wages	Median Price	Days of Wages
First Line Treatment, ACT for uncomplicated malaria	AL1	0.87	0.40	0.87	0.40	0.87	0.40	0.87	0.40
	AA1	1.19	0.55	1.52	0.70	1.30	0.60	1.30	0.60
	DHP	2.17	1.00	2.38	1.10	2.06	0.95	2.06	0.95
Artemisinin- based monotherapy for uncomplicated malaria	Artesunate tab	1.63	0.75	1.52	0.70	1.52	0.70	1.08	0.50
	Dihydroartemisinin tab	NA	NA	NA	NA	1.67	0.77	2.17	1.00
Non-artemisinin-based monotherapy for uncomplicated malaria	Chloroquine syrup	0.65	0.30	0.54	0.25	0.54	0.25	0.54	0.25
	Quinine tab	NA	NA	NA	NA	0.98	0.45	1.08	0.50
	Quinine Syrup	NA	NA	NA	NA	1.52	0.70	1.52	0.70
	Amodiaquine tab	0.65	0.30	0.70	0.33	0.76	0.35	0.76	0.35
Arteminisin based for severe malaria	Artesunate injection	1.08	0.50	1.08	0.50	1.08	0.50	1.08	0.50
	Artemether Inj	0.54	0.25	0.76	0.35	0.76	0.35	0.76	0.35
Non-artemisinin based for severe malaria	Chloroquine Inj	NA	NA	NA	NA	0.65	0.30	0.65	0.30
	Quinine Inj	NA	NA	NA	NA	0.38	0.18	0.38	0.18
Chemo- preventive	SP tab	0.65	0.30	0.76	0.35	0.54	0.25	0.76	0.35
	SPAQ1	NA	NA	NA	NA	0.43	0.20	0.65	0.30
	SPAQ2	NA	NA	NA	NA	0.43	0.20	0.54	0.25
Diagnostics Test	mRDT	NA	NA	NA	NA	0.43	0.20	0.43	0.20

#### Median Prices and affordability by type of outlet

Most of the products were more expensive (requires more days of wage) in pharmacies than in patent medicine stores, with a few exceptions, DHP (OB), chloroquine syrup (OB), and quinine injection (LPG). Two products (LPGs of chloroquine syrup and artesunate injection) require the same days of wage in both the pharmacies and patent medicine stores. All products except for the originator brand of DHP in patent medicine stores (days of wage = 1.2) were affordable (Table 8).

**Table 8.** Median prices and affordability in patent medicine stores and pharmacies.

Groups	Products	OB				LPG			
		PMS		Pharmacy		PMS		Pharmacy	
		Median Prices	Days of wages	Median Price	Days of Wages	Median Prices	Days of wages	Median Price	Days of Wages
First Line Treatment, ACT for uncomplicated malaria	AL1	0.87	0.40	1.08	0.50	0.87	0.40	1.03	0.48
	AA1	1.19	0.55	1.59	0.74	1.03	0.48	1.73	0.80
	DHP	2.60	1.20	2.17	1.00	1.73	0.80	2.06	0.95
Artemisinin- based monotherapy for uncomplicated malaria	Artesunate tab	1.08	0.50	1.95	0.90	1.08	0.50	1.52	0.70
	Dihydroartemisinin tab	NA	NA	NA	NA	1.95	0.90	2.17	1.00
Non-artemisinin-based monotherapy for uncomplicated malaria	Chloroquine syrup	0.65	0.30	0.54	0.25	0.54	0.25	0.54	0.25
	Quinine tab	NA	NA	NA	NA	0.65	0.30	1.08	0.50
	Quinine syrup	NA	NA	NA	NA	1.41	0.65	1.73	0.80
	Amodiaquine tab	0.54	0.25	1.08	0.50	0.65	0.30	1.30	0.60
Arteminisin based for severe malaria	Artesunate inj	1.08	0.50	1.19	0.55	1.08	0.50	1.08	0.50
	Artemether inj	0.65	0.30	0.76	0.35	0.76	0.35	0.78	0.36
Non-artemisinin based for severe malaria	Chloroquine inj	NA	NA	NA	NA	0.61	0.28	0.65	0.30
	Quinine inj	NA	NA	NA	NA	0.43	0.20	0.33	0.15
Non-artemisinin-based combination therapy	SP tab	0.54	0.25	0.76	0.35	0.54	0.25	0.76	0.35
	SPAQ1	NA	NA	NA	NA	0.33	0.15	1.30	0.60
	SPAQ2	NA	NA	NA	NA	0.43	0.20	0.54	0.25
Diagnostics Test	mRDT	NA	NA	NA	NA	0.43	0.20	1.08	0.50

## Prices and affordability of ACT + mRDT following WHO recommendations of malaria diagnosis before treatment

The overall median price for mRDT and ACTs ranged from USD 1.41 to USD 3.32. All other product combinations were affordable except for the OB and LPG of DHP and mRDT (days of wage of 1.53 and 1.52, respectively) (Table 9). The cost of OB and LPG of mRDT and DHP remained unaffordable across rural and urban locations, as well as pharmacies and patent medicines stores. Also, the OB and LPG of mRDT and AL1 as well as mRDT and AA1 were unaffordable in pharmacies.

**Table 9.** Median prices and affordability of recommended ACT and mRDT.

Group	Commodity type	Median price of ACT + mRDT (USD)			Affordability of ACT + mRDT (Days of wage)		
		AL1 + mRDT	AA1 + mRDT	DHP + mRDT	AL1 + mRDT	AA1 + mRDT	DHP + mRDT
Overall	OB	1.41	1.84	3.32	0.65	0.85	1.53
	LPG	1.41	1.66	3.28	0.65	0.77	1.52
Rural	OB	1.30	1.79	3.47	0.60	0.83	1.60
	LPG	1.46	1.95	3.25	0.68	0.90	1.50
Urban	OB	1.41	1.84	3.30	0.65	0.85	1.53
	LPG	1.41	1.66	3.32	0.65	0.77	1.53
PMS	OB	1.30	1.50	3.26	0.60	0.69	1.50
	LPG	1.30	1.14	3.25	0.60	0.53	1.50
Pharmacy	OB	3.90	3.90	6.93	1.80	1.80	3.20
	LPG	4.12	5.44	6.93	1.90	2.51	3.20

## Discussion

The overall private sector availability of under-five malaria commodities in surveyed locations was suboptimal. Artemisinin-based antimalarials for uncomplicated and severe malaria were the most available commodities. However, only about half (48.3% and 53.3%) of the surveyed outlets had stock of artemether/lumefantrine (AL1) and artesunate injection, respectively. The most available chemopreventive agent was the originator brand of sulphadoxine/pyrimethamine, with an availability of 27.3%, while mRDT was available in 37.2% of visited outlets. Across all products, the originator brand was more available than the lowest-priced generic. Except for a few products, availability for most products was higher in the urban areas compared to rural areas and in pharmacies compared to patent medicine stores. The median price of surveyed products ranged from USD 0.38 to USD 2.17 per treatment/test. Except for amodiaquine tablet and artemether injection, which cost less, all other originator brands cost the same or more than the lowest-priced generic. Most products were more expensive in urban areas and pharmacies. Antimalarial products were affordable as their median prices were not more than a day’s wage for the least-paid government worker. However, when the cost of testing and treatment with artemisinin-based combination therapies (ACTs) was assessed, testing and treatment with dihydroartemisinin/piperaquine were unaffordable as they cost more than 1.5 times the daily wage of the least-paid government worker across all locations and type of outlet

In line with the findings from this study (Edwards et al., 2022b; Ezenduka et al., 2013), a study conducted in 2021 also revealed that the availability of WHO-prequalified ACTs is decreasing in African countries like Nigeria and does not meet the World Health Organization’s mean availability benchmark of 80% for essential medicines (Jha et al., 2022; Mogojwe, 2022; World Health Organization, 2023). Decreasing availability could be attributed to many factors, especially COVID-19 pandemic, which significantly disrupted the global economy and supply chain (Kazancoglu et al., 2023; Moosavi et al., 2022).

The availability of monotherapies for malaria treatment such as dihydroartemisinin and chloroquine in some visited outlets, suggests that monotherapies may still be in use for malaria treatment in Nigeria, contrary to WHO’s recommendation of using ACTs. These products are generally cheaper than ACTs and may be attractive to low-income earners. The use of these monotherapies for malaria can increase the chances of the development of resistant strains and treatment failure, constituting a potential threat to optimal health outcomes. However, it is noteworthy that some individuals may purchase a combination of two monotherapies in these outlets, for example, artesunate tablet and SP.

The median prices of surveyed products are higher than in earlier years and may potentially be linked to the end of the AMFm and PSCM projects, where quality-assured malaria medicines were subsidised (Ebere Emilia et al., 2021; Ezenduka et al., 2013; Ezenduka et al., 2014), or COVID-19 pandemic, which has been suggested to be responsible for more than a 50% rise in the price of essential medicines in Nigeria (Emmanuel Awucha et al., 2020). A study after the subsidy project suggests the continued impact of the project on prices of ACTs (Akulayi et al., 2017) thus COVID 19 pandemic may be a major contributing factor to the higher prices observed in this study.

Overall, all products were affordable as they cost less than a day’s wage for the least-paid government worker (Raju, 2019). However, the cost of testing and treatment which is recommended for all of cases fever was not affordable across all product combinations, locations and types of outlets (World Health Organization, 2022b). This may result in treating malaria without a confirmatory test, resulting in the development of resistance to malaria medicines, which may further increase the burden of malaria. Hence, government interventions to improve affordability may be required if the target of reducing malaria mortality rates by at least 90% by 2030, as captured in the WHO Global Technical Strategy for Malaria 2016–2030 is to be met (Lee et al., 2021; World Health Organization, 2021)

Product prices were higher in pharmacies compared to patent medicine stores, potentially due to higher markups in the former. However, contrary to expectations, product prices in rural areas appear lower than in urban areas, suggesting better affordability despite the expected higher logistical cost for rural locations (Lee et al., 2021). This may be due to lower markup by rural outlet owners, given the expected lower earnings of rural dwellers. It could also be that fake or counterfeit products which would be cheaper are being stocked by rural outlets as there is limited regulatory oversight partly due to poor accessibility. There is, therefore an urgent need to investigate the quality of essential medicines in rural locations to safeguard the health of populace.

### Study limitations

First, we only focused on two dimensions of access to medicines, availability and affordability and did not evaluate the other three dimensions, including quality, accessibility and acceptability (Wirtz et al., 2016). Second, our assessment of affordability is only limited to the number of income days required to purchase malaria products. We could not the compute median price ratio (MPR) based on international reference prices as some of the malaria commodities included in this study were not captured in the international buyers’ reference price list maintained by Management Sciences for Health (MSH). Also, the latest version of the MSH’s reference price list, which is generally used as a reference price source was last updated in 2015, making it too old to be used for this study. Finally, our study is not generalisable to the entire country as we only included private outlets in states and the FCT where Malaria Consortium is implementing SMC campaigns.

## Conclusion

Our study indicates that the overall availability of under-five malaria commodities in the private sector in Nigeria is suboptimal. Although, malaria treatment alone was affordable, diagnosis and treatment were not affordable across all product combinations, locations and types of outlets as they cost more than a day’s wage for the least-paid government worker. Thus, suggesting the need for interventions, which may include subsidy initiatives, especially in rural areas, to reduce malaria burden and achieve universal health coverage.
